# Streptavidin-Conjugated DNA for the Boronate Affinity-Based Detection of Poly(ADP-Ribose) Polymerase-1 with Improved Sensitivity

**DOI:** 10.3390/bios13070723

**Published:** 2023-07-10

**Authors:** Fengli Gao, Gang Liu, Yishu Qiao, Xiuwen Dong, Lin Liu

**Affiliations:** 1 College of Chemistry and Chemical Engineering, Anyang Normal University, Anyang 455000, China; 2 College of Chemistry and Chemical Engineering, Henan University of Technology, Zhengzhou 450001, China

**Keywords:** streptavidin, nitrilotriacetic acid, magnetic bead, poly(ADP-ribose) polymerase, boronic acid, immobilization-free

## Abstract

This work reports the development of a fluorescence method for the detection of poly(ADP-ribose) polymerase-1 (PARP1), in which a phenylboronic acid-modified fluorescein isothiocyanate dye (FITC-PBA) was used to recognize the formed poly(ADP-ribose) (PAR) polymer. The detection system was designed by conjugating recombinant streptavidin (rSA) with PARP1-specific double-stranded DNA (dsDNA) through streptavidin–biotin interaction. Capture of PARP1 via rSA–biotin–dsDNA allowed for the poly-ADP-ribosylation (PARylation) of both rSA and PARP1 in a homogeneous solution. The resulting rSA–biotin–dsDNA/PAR conjugates were then captured and separated via the commercialized nitrilotriacetic acid–nickel ion-modified magnetic bead (MB-NTA-Ni) through the interaction between NTA–Ni on MB surface and oligohistidine (His_6_) tag in rSA. The PAR polymer could capture the dye of FITC-PBA through the borate ester interaction between the boronic acid moiety in PBA and the *cis*-diol group in ribose, thus causing a decrease in fluorescence signal. The PARylation of streptavidin and the influence of steric hindrance on PARylation efficiency were confirmed using reasonable detection strategies. The method showed a wide linear range (0.01~20 U) and a low detection limit (0.01 U). This work should be valuable for the development of novel biosensors for the detection of poly(ADP-ribose) polymerases and diol-containing species.

## 1. Introduction

Poly(ADP-ribose) polymerase-1 (PARP1) is a mammalian enzyme that ensures an essential function in maintaining genomic stability by regulating DNA repair and transcription [1]. The enzyme can be activated by binding to DNA with the structures of single- and double-strand breaks, hairpins, cruciforms or stably unpaired regions [2,3,4]. The activated PARP1 can trigger the poly-ADP-ribosylation (PARylation) of target proteins at the amino acid residues of serine, glutamate, aspartate, lysine and tyrosine, including histone, transcription factor and PARP1 itself. PARP1 is of interest for the diagnosis of ovarian, breast and oral cancers, as well as diseases caused by oxidative damage, ischemic diseases, cardiac hypertrophy, diabetes, inflammation or neuronaldeath [5]. The highly expressed PARP1 has been regarded as a potential biomarker and a therapeutic target for some diseases [5,6,7]. Therefore, it is of great importance to develop sensitive and selective methods for the detection of PARP1 activity.

The PARylation of PARP1 from nicotinamide adenine dinucleotide (NAD^+^) can cause the formation of poly(ADP-ribose) (PAR) polymers with linear and branched chains comprising up to 200 ADP-ribose units. The early methods for the assays of PARP1 activity are usually conducted with the use of biotin- or radio-labeled NAD^+^ analogues [8,9,10]. These methods are feasible but have the shortcomings of high cost and complex synthesis of substrate. By monitoring the consumption of NAD^+^ substrates, the commercial NAD/NADH-Glo™ kit can be used for probing into PARP1 activity; however, this method involves the use of additional enzymes, and endogenous NAD/NADH in biological samples will interfere with the activity assays. For this reason, a series of novel methods have been developed for PARP1 detection by monitoring the produced PAR polymers through electrostatic interactions between the negatively charged ribose units and the positively charged signal molecules [11,12,13,14,15,16,17,18,19,20,21,22,23,24]. For example, Dai and co-workers designed a reusable electrochemical biosensor with positively charged hexaammineruthenium(III) chloride (RuHex) to recognize the resulting PAR polymers [25]. Wei’s group reported a series of electrochemical, colorimetric and fluorescent biosensors for PARP1 detection through electrostatic interactions [11,13,14,18,19,20,21,22,23,24]. The resulting PAR polymers could also be determined viaquartz crystal microbalance (QCM) with positively charged gold nanorods for signal amplification [12]. All of the heterogeneous methods are sensitive and do not require the use of labeled NAD^+^ analogues. However, they have the disadvantages of false positive signals caused by the electrostatic interactions between signal reporters and DNA probes, the tedious process for sensor fabrication, and the low PARylation efficiency due to steric hindrance [26,27]. In addition, the sensitivity of such heterogeneous methods may be disputed because the auto-modified PARP1 can be dissociated from the DNA-modified sensing interface by steric and electrostatic repulsion [28,29].

In addition to the phosphate groups, many *cis*-diol groups are included in the ribose units of PAR polymers. Phenylboronic acid (PBA) can react with *cis*-diol group through the formation of cyclic borate ester covalent bond. Such an interaction has allowed for the recognition and separation of *cis*-diol-containing biomolecules [30,31,32,33,34]. More interestingly, several groups have achieved the detection of *cis*-diol-containing biological macromolecules using boronic acid-functionalized derivatives or nanomaterials as the recognition elements [33,35,36,37,38,39,40,41,42,43,44,45,46,47,48,49,50,51]. For example, our group employed PBA-modified gold nanoparticles as the linkers to recognize glycoproteins and microRNAs and to conjugate electroactive signal reporters through the formation of borate ester covalent bonds [35,36]. Hu and co-workers reported the electrochemical detection of glycoproteins (mucin 1 and α-fetoprotein) and lipopolysaccharide with (4-(ferrocenylacetamido)-phenyl)boronic acid (FcPBA) as the signal probe to recognize the target attached on the sensor electrode [37,38,39]. Meanwhile, they achieved the detection of a BRCA1 breast cancer gene-derived DNA target using Zr(IV) ions as the linkers to decorate polysaccharide chains for coupling of FcPBA probes [40]. Tang’s group achieved the detection of live bacteria through the complexation of boronic acid-derived aggregation-induced emission fluorogens with *cis*-diols on the bacterial surface [46]. Wang’s group reported the fluorescent identification of glycoproteins and cancer cells using boronic acid-decorated carbon dots and carbon nitride nanosheets [44,45]. In this work, we attempted to investigate the PARylation and achieve the detection of PARP1 through the interaction between boronic acid and *cis*-diol in PAR polymer.

Streptavidin (SA) is an extremely stable protein which can bind with biotin or biotinylated molecules with high binding affinity (K_d_ = ~10^15^ M). Biotinylated antibodies/antigens and SA-modified plates, columns, nanomaterials and magnetic beads are commercially available in a variety of fields [52,53,54,55]. Recent studies reveal that SA conjugated with DNA can serve as a noncanonical substrate of PARylation due to the close proximity between PARP1 and SA [28], in which the PARylation of DNA-conjugated SA and the formation of PAR polymers were confirmed via ATR-FTIR. Herein, a PBA-modified dye of fluorescein isothiocyanate (FITC-PBA) was used to monitor the formation of PAR polymers. In contrast to the electrostatic interactions with positively charged signal molecules, the borate ester interaction can eliminate false positive signals since the FITC-PBA probe shows no interaction with DNA. In addition, SA molecules conjugated with DNA probes could also be poly(ADP-ribosyl)ated (PARated) by the activated PARP1. This can avoid the deficiencies of heterogeneous assays induced by steric hindrance on the PARylation efficiency and the dissociation of auto-modified PARP1 from DNA, thus improving the detection sensitivity.

## 2. Materials and Methods

### 2.1. Chemicals and Reagents

Nitrilotriacetic acid–nickel ion-modified magnetic bead (MB-NTA-Ni) was obtained from Thermo Fisher Scientific (Shanghai, China). FITC-PBA and SGHDEVDK-dansyl were synthesized and purified by ChinaPeptide Co., Ltd. (Shanghai, China). Triethylene glycol mono-11-mercaptoundecyl ether (HSC_11_PEG_3_-OH), bovine serum albumin (BSA), thrombin, NAD^+^ and NADP^+^ were purchased from Sigma-Aldrich Co., Ltd. (Shanghai, China). Hexaethylene glycol mono-11-mercaptoundecyl acid (HSC_11_PEG_6_-COOH) was obtained from Sensopath Technologies (Bozeman, MT, USA). PARP1 was ordered from AmyJet Scientific Inc. (Wuhan, China). PARP1 ELISA kit was obtained from KeboruiBiotech. Co., Ltd. (Shanghai, China). Avidin, glucose, DNA, SA and rSA were provided by Sangon Biotech. Co., Ltd. (Shanghai, China). Cetyltrimethylammonium bromide (CTAB)-coated gold nanorods (GNRs) were ordered from XFNANO Materials Tech. Co., Ltd. (Nanjing, China). Other reagents were ordered from Aladdin Reagent Co., Ltd. (Shanghai, China). All solutions were prepared with ultrapure water treated using a Millipore Milli-Q water system.

Biotin–dsDNA stock solution was prepared by mixing 20 μM biotinylated ssDNA (biotin-ssDNA, biotin-5′-CGA GTC TAC AGG GTT GCG GCC GCT TGG G-3′) and 25 μM complementary sequence (ssDNA, 5′-CCC AAG CGG CCG CAA CCC TGT AGA CTC G-3′) at 37 °C for 1 h in a TNE buffer (pH 7.4, 20 mM Tris-HCl and 0.1 M NaCl). To ensure that the amount of free biotin-ssDNA is negligible, the concentration of ssDNA was slightly higher than that of biotin-ssDNA.

### 2.2. Mass Spectrometry

NADP^+^ was dissolved in ultrapure water and FITC-PBA was dissolved in methanol. Then, 0.1 mL of 1 mM NADP^+^ was mixed with 0.1 mL of 1 mM FITC-PBA. After reaction for 5 min, 0.2 mL of the mixture was diluted to 1 mL with ultrapure water, and the mass spectrum was recorded on a LCT Premier XE mass spectrometer (Waters, Milford, MA, USA) with a negative ion mode.

### 2.3. Procedures for PARP1 Detection

**Scheme 1:**
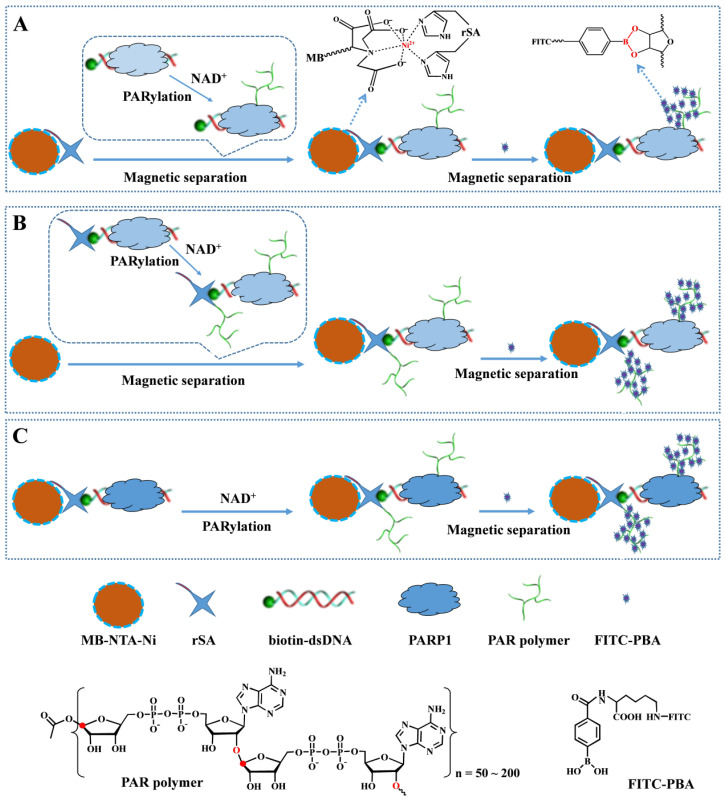
Schematic illustration of the proposed strategies for PARP1 detection based on the borate ester interaction: (**A**) immobilization-free PARylation of PAPR1, (**B**) immobilization-free PARylation of both PAPR1 and rSA, and (**C**) heterogeneous PARylation of both PAPR1 and rSA.

For Scheme 1A, 10 μL of biotin–dsDNA stock solution was added to 50 μL of reaction buffer containing a certain concentration of PARP1 and 500 μM NAD^+^. After reaction for 1 h at 37 °C, 40 μL of 0.5 mg/mL MB-NTA-Ni suspension containing 5 μM rSA was added to the reaction solution after 10 min of incubation. After being washed twice with the reaction buffer under magnetic separation, the resulting magnetic precipitates were exposed to 200 μL of 1 μM FITC-PBA in phosphate buffer (pH 7.4, 10 mM). After incubation for 5 min and treatment using a magnet, the supernatant solution was taken out for fluorescence analysis.

For Scheme 1B, 10 μL of biotin–dsDNA stock solution was added to 50 μL of reaction buffer containing a certain concentration of PARP1, 5 μM rSA and 500 μM NAD^+^. To evaluate inhibition efficiency, AG014699 at a given concentration was pre-incubated with PARP1 for 10 min. After reaction for 1 h at 37 °C, 40 μL of 0.5 mg/mL MB-NTA-Ni suspension was added to the reaction solution for 10 min of incubation. Other treatment and detection procedures were the same as those of Scheme 1A.

For Scheme 1C, 10 μL of biotin–dsDNA stock solution was mixed with 40 μL of 0.5 μg/mL MB-NTA-Ni suspension containing 5 μM rSA. After incubation for 10 min, 50 μL of reaction buffer containing a certain concentration of PARP1 and 500 μM NAD^+^ was added to the suspension. After reaction for 1 h at 37 °C and then washing twice under magnetic separation, the resulting magnetic precipitates were exposed to the FITC-PBA solution. The suspension was treated using a magnet and the supernatant liquid was measured using the procedures as those shown in Scheme 1A.

### 2.4. Probing of PARP1Activity via Surface Plasmon Resonance (SPR)

Gold-coated glass slides were used as the SPR chips. The chips were modified with HSC_11_PEG_6_-COOH and HSC_11_PEG_3_-OH by Au-S interaction for immobilization of SA proteins via a standard amino coupling reaction [56]. The mixed PEG self-assembled monolayer (SAM) was formed by incubation of gold chip with the mixture of 0.1 mM HSC_11_PEG_6_-COOH and 0.9 mM HSC_11_PEG_3_-OH in the dark for 48 h. After being washed with ethanol and purified water and then dried with nitrogen, the chips were incubated with the mixture of 100 mM EDC with 50 mM NHS for 15 min. The activated chips were then rinsed with water and incubated with 10 μM SA solution for 4 h. SA proteins were conjugated on the SAM using the standard amine coupling reaction. The unreacted sites were blocked by incubating the chips with 1 mM ethanolamine for 30 min.

To monitor the PARylation of both SA and PARP1 on the chip surface, SA-covered chip was incubated with 1 μM biotin–dsDNA solution for 30 min. After being washed with water, the chip was incubated with the reaction buffer containing 50 U PARP1 and 500 μM NAD^+^. After reaction for 30 min again, the chip was rinsed with water and then mounted onto the BI-SPR 3000 system (Biosensing Instrument Inc., Tempe, AZ, USA). When a stable baseline was obtained, the GNRs suspension was injected into the channel and the signal was recorded. To verify the PARylation of SA in this process, the PARP1/NAD^+^-treated sensor chip was incubated with 12.5% (*v*/*v*) phenol at room temperature for 30 min and then rinsed with 10 mM NaOH. This step can release biotin–dsDNA and PARP1 from the SA-covered chip [57]. Then, the chip was mounted onto the SPR instrument for the injection of GNRs.

### 2.5. Extraction of Cytoplasms

The cytoplasm samples were prepared using the procedures described in previous reports [24,58]. Briefly, MCF-7 cells were incubated in Dulbecco’s modified Eagle’s medium (DMEM) with 10% fetal bovine serum under a humidified atmosphere containing 5%CO_2_ at 37 °C. To induce the apoptosis, the cells were incubated with STS for 8 h. After being washed with phosphate buffer, living and apoptotic cells were collected and then treated with the cytoplasmic protein extraction reagent under vigorous shaking. This was followed by centrifugation at 11,000 rpm for 10 min at 4 °C. The supernatant cytoplasms were stored at −80 °C for use. Before the assays, the cytoplasms were diluted different times and the levels of PARP1 in the diluted cytoplasms were determined with the procedures as mentioned above.

### 2.6. Detection of Caspase-3 Activity in Cytoplasm Samples

To verify the STS-induced apoptosis, a peptide probe with Cu^2+^ as the quencher and dansyl as the fluorophore (Cu^2+^-SGHDEVDK-dansyl) was used to monitor the activity of caspase-3. The probe was prepared as presented in our previous report [59]. For the assays of caspase-3 in cytoplasm samples, 100 μL of probe in HEPES buffer was incubated with 100 μL of the diluted cytoplasm at 37 °C for 1 h. The fluorescence intensity was determined with an emission wavelength at 552 nm.

## 3. Results and Discussion

### 3.1. Strategies for PARP1 Detection

To monitor the PARylation of DNA-conjugated SA, recombinant SA (rSA) was used since it can be readily captured via commercialized MB-NTA-Ni through the NTA–Ni–oligohistidine interaction [60]. In the first trial, PARP1 was activated by binding to biotin–dsDNA and then PARated to form biotin–dsDNA/PAR conjugates (Scheme 1A). The resulting conjugates were separated via rSA-captured MB-NTA-Ni (denoted as MB-NTA-Ni-rSA) through the streptavidin–biotin interactions. The PAR polymers on the MB surface could capture a large number of FITC-PBA dyes through the borate ester interactions between the boracic acid groups in dyes and the *cis*-diol moieties in ribose units, thus causing the decrease in the number of free FITC-PBA molecules and the fluorescence intensity of solution. In the second trial, rSA-linked biotin–dsDNA (rSA–biotin–dsDNA) was used to bind and activate PARP1 (Scheme 1B). In this strategy, both rSA and PARP1 would be PARated. The resulting rSA–biotin–dsDNA/PAR conjugates were then captured and separated via MB-NTA-Ni through the interaction between the NTA–Ni complex on the MB surface and the oligohistidine (His_6_) tag in rSA. By measuring the difference in the fluorescence signal change of these two trials, the PARylation of rSA could be confirmed. To evaluate the effect of steric hindrance on PARylation efficiency, rSA–biotin–dsDNA was immobilized on the surface of MB-NTA-Ni and then PARP1 was captured and activated by binding to the dsDNA (Scheme 1C). In the presence of NAD^+^, both rSA and PARP1 anchored on the MB surface would be PARated. The PARylation efficiency was evaluated by comparing the fluorescence signal change with that achieved using the immobilization-free strategy, as shown in Scheme 1B.

### 3.2. Feasibility for PARP1 Detection

**Figure 1:**
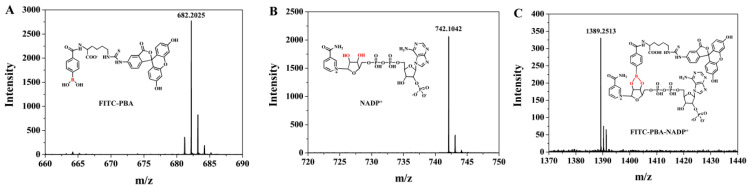
Mass spectra of FITC-PBA (**A**), NADP^+^ (**B**) and FITC–PBA–NADP^+^ (**C**).

Mass spectrometry is a viable measure that can provide direct evidence for the binding stoichiometry. In previous studies, boronic acids have been employed as derivatization reagents to improve the selectivity and sensitivity of *cis*-diol-containing metabolites for mass spectrometry analysis [58,61,62]. To explore the interaction between FITC-PBA probe and ADP ribose unit in the PAR polymer, the formation of FITC–PBA–NADP^+^ complex was first confirmed via mass spectrometry. As shown in Figure 1A,B, the dominant mass peak for FITC-PBA and NADP^+^ with one negative charge is 682.2025 Da and 742.1042 Da, respectively. The mixture of FITC-PBA and NADP^+^ shows a dominant mass peak at 1389.2513 Da, corresponding to that of FITC–PBA–NADP^+^ with one negative charge. The m/z is consistent with the calculated value, indicating the formation of borate ester bond between *cis*-diol in ADP ribose unit and boronic acid group in FITC-PBA.

**Figure 2:**
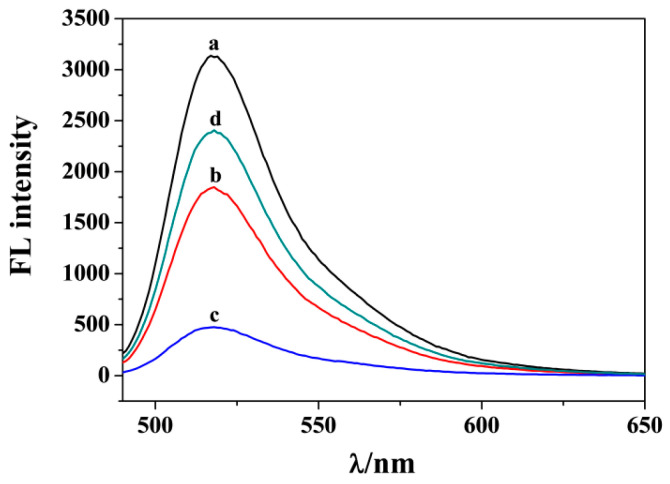
Fluorescence emission spectra of 1 μM FITC-BA before (curve a) and after treatment using the PAR-covered MB conjugates obtained via different schemes: Scheme 1A/curve b, Scheme 1B/curve c, and Scheme 1C/curve d. The concentration of PARP1 was 50 U.

PARP1 can be captured and activated by binding to DNA with a given structure. The activated PARP1 can trigger the PARylation of various proteins. Recent studies suggested that SA in a close proximity to the activated PARP1 can serve as a noncanonical substrate of PARylation [28]. Herein, PARylation of rSA was confirmed by monitoring the formation of PAR polymers in rSA and PARP1 with FITC-PBA as the signal probe. The probe can react with the *cis*-diol groups in the ribose units of PAR polymers through the borate ester interactions. As shown in Figure 2, the solution of FITC-PBA shows high fluorescence at 520 nm (curve a). After incubation with the biotin–dsDNA/PAR conjugates-covered MB-NTA-Ni-rSA (Scheme 1A), the fluorescence signal of FITC-PBA decreased (curve b), indicating that the probe could be captured and removed by binding to the PAR polymers on the MB surface. When PARP1 was activated by the rSA–biotin–dsDNA conjugate and then separated via MB-NTA-Ni (Scheme 1B), a more significant decrease in the fluorescence signal was observed (curve c). The result implied that rSA was also PARated, thus sequestering a greater number of FITC-PBA dyes. To investigate the influence of steric hindrance on the PARylation efficiency, rSA–biotin–dsDNA was immobilized on the surface of MB-NTA-Ni for the capture and activation of PARP1 (Scheme 1C). The resulting PAR polymers in both rSA and PARP1 on the MB surface also caused the decrease in fluorescence signal (curve d). However, the fluorescence intensity was higher than that obtained using the immobilization-free method (curve c), indicating that PARylation efficiency was limited due to steric hindrance. All of the results demonstrated that the proposed schemes could be used to monitor PARP1 activity by sequestering FITC-PBA dye to quench the fluorescence signal through the borate ester interaction. We also found that the MB-NTA-Ni and FITC-PBA probe exhibited high stability after storage at 4 °C for at least six months.

### 3.3. SPR Analysis

**Figure 3:**
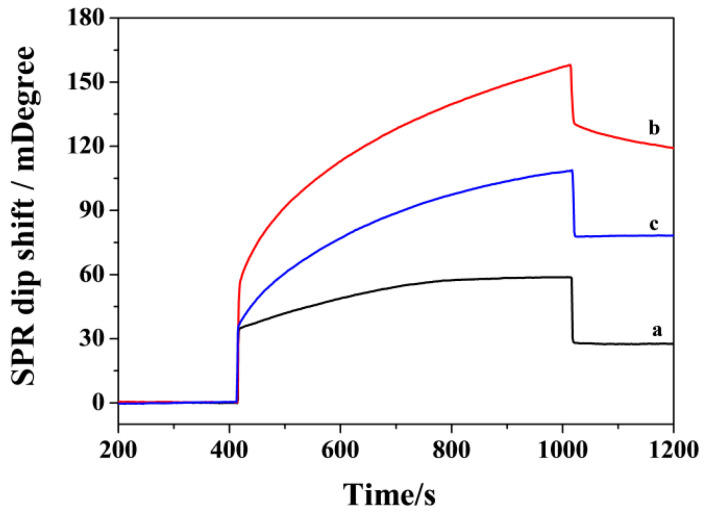
SPR sensorgrams when injecting GNRs to the SA-covered chip before curve a and after curve b treatment with biotin–dsDNA/PARP1 for the PARylation. Curve c corresponds to that when injecting GNRs to the PARylated surface that has been treated by 12% (*v*/*v*) phenol.

SPR is a sensitive surface analysis technique that can monitor the change in the dielectric constant caused by molecular adsorption on heavy metal films. This method has been widely used in the study of biomolecular interactions. However, the conventional SPR methods are unable to detect a small change in refractive index. Previous investigations suggested that the positively charged nanomaterials could be used to distinguish the produced PAR polymer via electrostatic interactions. A typical example is that Yang and co-workers achieved the detection of PARP1 by discerning PAR effectively with the positively charged CTAB-coated GNRs to amplify the frequency change of the QCM biosensor [12]. Inspired by the result and the similar sensing principle of the SPR and QCM biosensors, we investigated the PARylation of both SA and PARP1 on the chip surface. SA was covalently immobilized on the chip surface via the amino coupling reaction. Then, biotin–dsDNA duplexes were attached onto the SA-covered chip for the capture of PARP1. After PARylation in the presence of NAD^+^, GNRs were injected into the channel to recognize the produced PAR polymers. As shown in Figure 3, SPR signal was significantly higher when injecting GNRs to the PARylated surface (curve b) than without the capture of biotin–dsDNA for the PARylation (curve a), indicating that the SPR method can also be used to monitor the formation of PAR polymer. To verify that that SA proteins attached on the chip surface were also PARated, biotin–dsDNA and PARP1 were released using 12% (*v*/*v*) phenol and then GNRs were injected into the channel. As a result, a decreased SPR signal was observed (curve c). However, the signal was still higher than that by injecting GNRs to the SA-covered chip, indicating that SA was also PARylated and the formed PAR polymer in SA facilitated the capture of GNRs.

### 3.4. Analytical Performances

**Figure 4:**
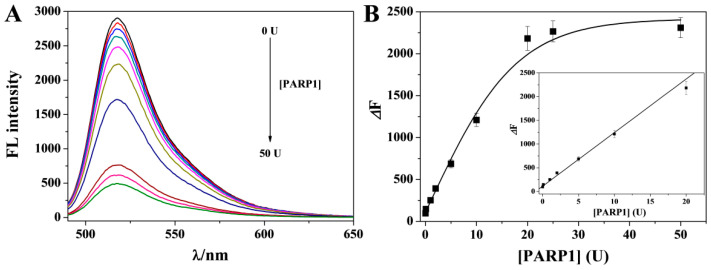
(**A**) Fluorescence emission spectra for the detection of different concentrations of PARP1 (from top to bottom: 0, 0.01, 0.1, 1, 2, 5, 10, 20, 25 and 50 U). (**B**) Plots of ΔF against PARP1 concentration.

To evaluate the analytical performances of the method, different concentrations of PARP1 was determined with the proposal of Scheme 1B in view of its high sensitivity and simplicity. As shown in Figure 4A, the fluorescence signals decreased gradually when the rSA–biotin–dsDNA conjugates were incubated with increasing concentration of PARP1 and then separated via MB-NTA-Ni. Thus, a higher concentration of PARP1 can permit the generation of more PAR polymers on the MB surface, thus sequestrating more FITC-PBA probes. No significant change in the fluorescence signal was observed in the absence of PARP1, indicating that the dye of FITC-PBA showed no interaction with rSA–biotin–dsDNA and MB-NTA-Ni. The fluorescence intensity change (ΔF = F_0_ − F_1_, where F_0_ and F_1_ represent the fluorescence intensity of the system in the absence and presence of PARP1, respectively) was used to evaluate the performances of the method. As shown in Figure 4B, ΔF increased linearly with the increase in PARP1 concentration and then began to level off beyond 20 U. The platform is indicative of the achievement of PARylation. The relative standard deviations (RSDs) were all less than 8.6%, which is indicative of good reproducibility of this method. The linear equation was found to be ΔF = 112 + 113[PARP1] (U) with a lowest detectable concentration of 0.01 U. The value is comparable to that obtained via heterogeneous methods through electrostatic interactions (Table 1). The high sensitivity can be attributed to the high PARylation efficiency and the specific borate ester interaction.

### 3.5. Evaluation of Inhibition Efficiency

**Figure 5:**
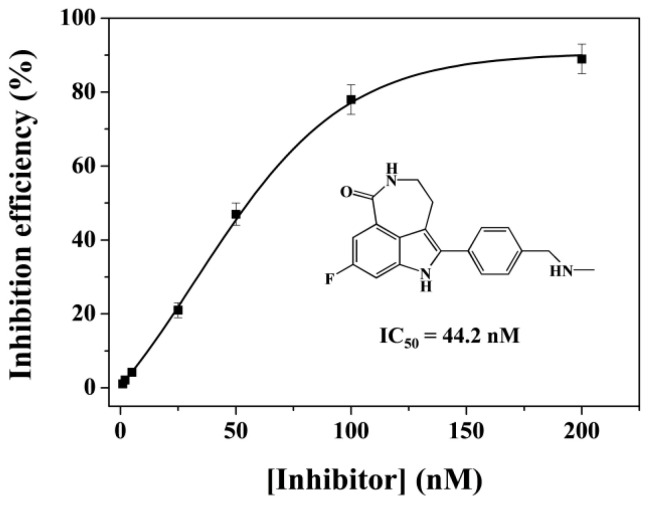
Inhibition efficiency of different concentrations of AG014699 for 10 U PARP1.

To investigate the inhibition efficiency, PARP1 was incubated with various concentrations of AG014699 (a well-known PARP1 inhibitor) and then analyzed with Scheme 1B. After extraction via MB-NTA-Ni and separation using a magnet, FITC-PBA was added to the magnetic precipitates. The inhibition efficiency was determined with the formula of inhibition (%) = 100 × (F_0_ − F_2_)/(F_0_ − F_1_), where F_2_ represents the fluorescence intensity in the presence of PARP1 with a given concentration of inhibitor. Figure 5 shows the dependence of inhibition efficiency on inhibitor concentration. It was found that the value was intensified with the increase in inhibitor concentration. Thus, a higher concentration of inhibitor can limit the PARylation more efficiently. The half maximal inhibitory concentration (IC_50_) value was found to be 44.2 nM for 10 U PARP1, which is consistent with that attained using other methods [58,63,65]. Thus, the proposed method could be used for evaluating the inhibition efficiency of potential PARP1 inhibitors with high simplicity and throughput.

### 3.6. Selectivity

**Figure 6:**
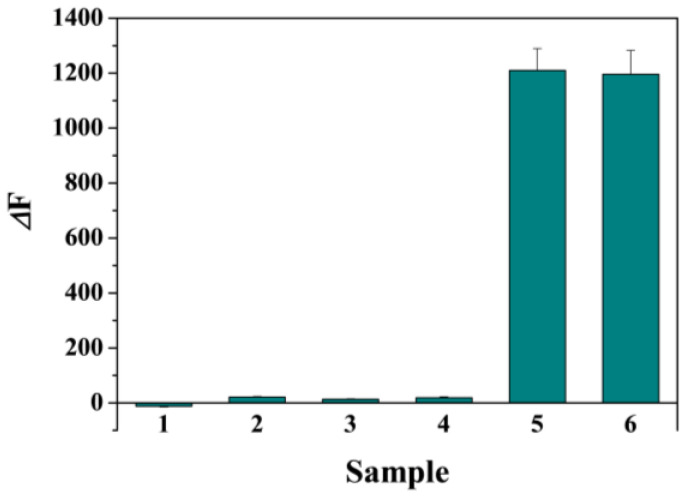
Selectivity. Bar 1, 0.1 mg/mL BSA; bar 2, 0.1 mg/mL avidin; bar 3, 0.1 mg/mL thrombin; bar 4, 1 mM glucose; bar 5, 10 U PARP1; bar 6, the mixture of 1~5.

To study the selectivity of this strategy, the sensing system was challenged with different biological species, such as serum albumin BSA (bar 1), glycoprotein avidin (bar 2), protease thrombin (bar 3) and small molecule glucose (bar 4). As a result, only PARP1 led to a significant change in fluorescence intensity (bar 5) (Figure 6). For BSA and thrombin, the results are accessible since the two proteins show no interaction with the FITC-PBA probe. Although avidin and glucose can react with boric acids to form borate ester bonds, the two diol-containing species did not significantly induce the signal change. This is understandable since they cannot be captured via MB-NTA-Ni through the metal–oligohistidine interactions. Therefore, although the FITC-PBA probes can also react with other intrinsic molecules containing *cis*-diol groups, the interferences from the intrinsic biological species can be easily eliminated during the magnetic separation step. In addition, we also found that the four tested biomolecules showed no interference in the assay of PARP1 activity (bar 6), indicating that the method shows high specificity and exhibits great potential to determine PARP1 in biological samples. Moreover, even if other species in biological matrixes may influence the detection of PARP1, the proposal of Scheme 1C could be performed to eliminate the potential interference by preconcentration of PARP1.

### 3.7. Real Sample Assays

**Figure 7:**
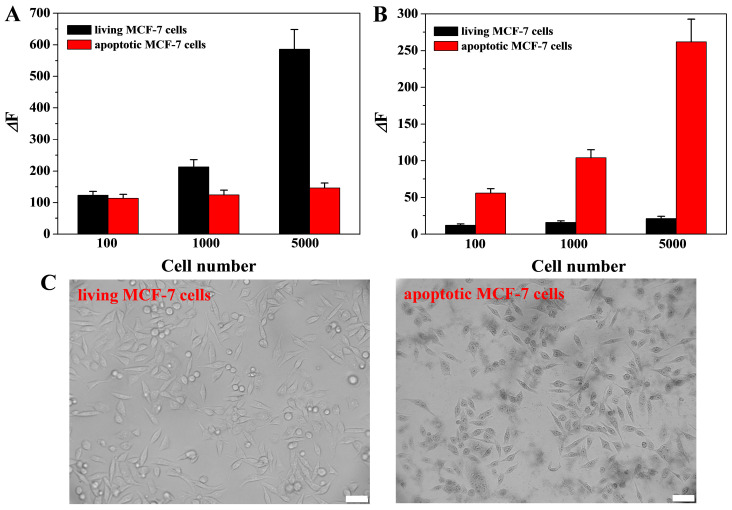
Results for the detection of PARP1 (**A**) and caspase-3 (**B**) in living and apoptotic cells. (**C**) Morphology images of normal and apoptotic MCF-7 cells. The scale bar in (**C**) is 50 μm.

The level or activity of PARP1 is related to many tumor and inflammation diseases. To probe into the application of the method for clinical analysis, the levels of PARP1 in the cytoplasms of living and apoptotic cells were determined. As depicted in Figure 7A, the signal change increased gradually with the increase in the number of living MCF-7 cells, indicating that the system could be used to determine PARP1 in the cytoplasm of MCF-7 cells. However, no significant change was found for the cytoplasm extracted from apoptotic MCF-7 cells. The result can be explained by the fact that apoptosis activated the activity of caspases, thus inducing the digestion of PARP1 into two segments and making it lost the ability of PARylation [66,67]. Among the caspase family, caspase-3, a central mediator for controlling internal and external apoptosis pathways, is acknowledged as the therapeutic target and diagnostic biomarker for apoptosis-relative diseases. The peptide containing a sequence of Asp-Glu-Val-Asp (DEVD) can be specifically recognized and cleaved by caspase-3 at the C-terminus. To verify the apoptosis, a peptide probe of SGHDEVDK-dansyl was used to monitor the activity of caspase-3 [59]. As shown in Figure 7B, the fluorescence intensity change increased gradually when the cells were treated by the inducer STS, while no significant increase was observed for the analysis of cytoplasm extracted from living cells. The results demonstrated that caspase-3 in the cells was indeed activated during apoptosis. The result was also confirmed by the morphology change of the living and apoptotic cells (Figure 7C). Therefore, the presented method for monitoring PARP1 activity shows promising applications in evaluating cell apoptosis and developing potential drugs for apoptosis-related diseases.

**Figure 8:**
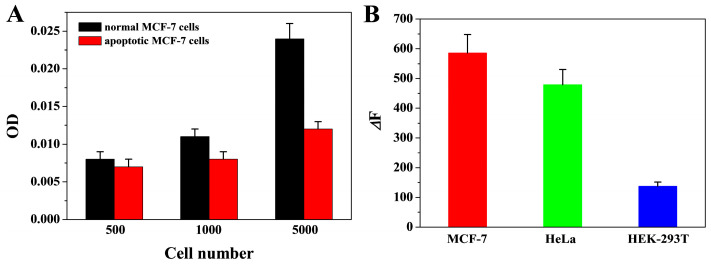
(**A**) Dependence of OD value on the number of normal and apoptotic MCF-7 cells. (**B**) Results for the assays of PARP1 in 5000 living MCF-7, HeLa and HEK-293 cells.

To demonstrate that PARP1 was digested during cell apoptosis, we monitored the change in PARP1 concentration in both living and apoptotic MCF-7 cells using a commercial ELISA kit. The optical density (OD) values for living cells were significantly higher than those for apoptotic cells (Figure 8A), indicating that apoptosis induced the decrease in the level of endogenous PARP1. To further demonstrate the feasibility of this method for the assays of real biological samples, we monitored the activity of PARP1 in normal human embryonic kidney cell line (HEK-239T) and cervical carcinoma cell line (HeLa). As shown in Figure 8B, the change in fluorescence intensity induced by cancer cells (HeLa and MCF-7) were significantly higher than that by HEK-293T cells, indicating that the expression level of PARP-1 in cancer cells is higher than that in normal cells.

## 4. Conclusions

In summary, we suggested that SA could be conjugated with DNA to design biosensors for the detection of PARP1. The method did not require the pre-immobilization of the DNA probe on the solid surface for PARylation, thus improving the catalytic reaction efficiency. In addition, it was confirmed that SA with a close proximity to the captured PARP1 can also be PARated, thus allowing for the design of immobilization-free biosensors with improved sensitivity. In contrast to the previously reported methods, the proposed detection system was designed based on the formation of specific borate ester bonds but not the nonspecific electrostatic interactions, which can eliminate the false positive signals. The method was used to evaluate the inhibition efficiency of a classical inhibitor and measure the activity of PARP1 in living and apoptotic cells with satisfactory results. In addition, this work provides important information for the development of novel homogeneous biosensors for monitoring PARP1 activity and evaluating cell apoptosis.

## Figures and Tables

**Table 1:** Overview of the detection of PARP-1 via different methods.

Method	Signal Reporter	Linear Range	Detection Limit	Ref.
photoelectrochemistry	PFP	0.01~2 U	0.007 U	[11]
QCM	CTAB-GNRs	0.06~1.2 nM	0.04 nM	[12]
colorimetry	NAD-AuNPs	0.43~1.74 nM	0.32 nM	[16]
colorimetry	CTAB-GNRs	0.05~1.0 U	0.006 U	[63]
colorimetry	hemin-graphene	0.05~1.0 U	0.003 U	[24]
chemiluminescence	AuNCs	0.01~1.0 U	0.009 U	[21]
electrochemistry	MBs/FcBA	0.1~50 U	0.1 U	[58]
electrochemistry	[Ru(NH_3_)_6_]^3+^	0.01~1 U	0.003 U	[25]
electrochemistry	Polyaniline	0.005~1.0 U	0.002 U	[15]
electrochemistry	Artificial nanochannels	0.05~1.5 U	0.006 U	[64]
electrochemistry	NH_2_-MSFs	0.01~1.2 U	0.005 U	[65]
electrochemistry	P-CuNPs	0.01~1U	0.004 U	[13]
electrochemistry	PMo_12_O_40_^3−^	0.01~1.0 U	0.008 U	[14]
fluorescence	PFP/MnO_2_	0.024~1.2 nM	0.003 nM	[22]
fluorescence	TOTO-1	0.02~1.5 U	0.02 U	[20]
fluorescence	FITC-PBA	0.01~20 U	0.01 U	This work

Abbreviations: PFP, poly[9,9-bis(6′-N,N,N-trimethylammonium)hexyl]fluorenylene phenylene; CTAB-GNRs, positively charged cetyltrimethylammonium bromide-coatedgold nanorods; AuNPs, gold nanoparticles; AuNCs, gold nanocluster; MBs, magnetic beads; FcBA, ferrocenylboronic acid; NH_2_-MSFs, positively charged amino-functioned mesoporous silica films; P-CuNPs, peptide-templated copper nanoparticles; TOTO-1, an unsymmetrical cyanine dye dimer.

## Data Availability

Not applicable.
